# Bariatric Conversion Surgery Impact on LDL Cholesterol in Patients Previously Treated with Sleeve Gastrectomy

**DOI:** 10.3390/jcm14144901

**Published:** 2025-07-10

**Authors:** David Benaiges, Max Calzada, Anna Casajoana, Belen Deza, Manuel Pera, Elisenda Climent, Juana A. Flores Le Roux, Marc Beisani, Miguel Olano, Karla A. Pérez-Vega, Juan Pedro-Botet, Albert Goday

**Affiliations:** 1 Department of Endocrinology and Nutrition, Hospital del Mar, Passeig Marítim, 25–29, 08003 Barcelona, Spainmariabelen.deza.castro@hmar.cat (B.D.); ecliment@hmar.cat (E.C.); jaflores@hmar.cat (J.A.F.L.R.); miguel.olano.arregui@hmar.cat (M.O.); karlaalejandra.perez.vega@hmar.cat (K.A.P.-V.); jpedrobotet@hmar.cat (J.P.-B.); agoday@hmar.cat (A.G.); 2 Department of Medicine, Universitat Pompeu Fabra, Plaça de la Mercè, 10–12, 08002 Barcelona, Spain; 3 Unit of Cardiovascular Risk and Nutrition, Institut Hospital del Mar d’Investigacions Mèdiques (IMIM), Dr. Aiguader, 80, 08003 Barcelona, Spain; acasajoanabadia@hmar.cat; 4 Consorci Sanitari de l’Alt Penedès i Garraf, 08720 Vilafranca del Penedès, Spain; 5 CiberOBN, Instituto de Salud Carlos III, Avenida Monforte de Lemos, 3–5, Pabellón 11, Planta 0, 28029 Madrid, Spain; 6 Esophago-Gastric and Bariatric Surgery Unit, Department of Surgery, Hospital del Mar, Passeig Marítim, 25–29, 08003 Barcelona, Spain; pera@hmar.cat (M.P.); mbeisani@hmar.cat (M.B.); 7 Department of Medicine, Universitat Autònoma de Barcelona, Plaça Cívica, Bellaterra, 08193 Barcelona, Spain

**Keywords:** bariatric surgery, conversional bariatric surgery, LDL cholesterol, single-anastomosis duodeno–ileal bypass with sleeve gastrectomy, sleeve gastrectomy, gastric bypass

## Abstract

**Background/Objectives**: Many patients with obesity require conversion bariatric surgery (CBS) after sleeve gastrectomy (SG). The objective of this study was to assess the evolution of LDL cholesterol and other cardiometabolic parameters in patients who have undergone an SG and require a CBS, as the metabolic effects of such conversion procedures remain insufficiently understood. **Methods**: A retrospective analysis was conducted in a non-randomized prospective cohort of patients with severe obesity who were previously treated with SG and undergoing CBS. Changes in LDL cholesterol levels after SG were compared to those following CBS using repeated-measures ANOVA. **Results**: Twenty-eight patients were included (mean age 44.5 ± 7.2 years; 68% female; mean BMI 47.3 ± 7.2 kg/m^2^). Of these, 57% underwent Roux-en-Y gastric bypass (RYGB), and 43% underwent single-anastomosis duodeno–ileal bypass with sleeve gastrectomy (SADI-S) as conversion procedures. The mean time between SG and CBS was 93.5 ± 45.3 months for RYGB and 31.0 ± 45.2 months for SADI-S. The change in LDL cholesterol pre- vs. post-SG was 3.3 mg/dL (95% CI: −13.6 to 20.1), whereas the change pre- vs. post-CBS was −25.7 mg/dL (95% CI: −37.5 to −13.9) (*p* < 0.001). Remission of high LDL-C was 18.8% after SG and 73.3% after CBS (*p* = 0.023). The cardiometabolic profile showed a marked improvement profile during the SG period, followed by maintenance of these improvements during the CBS period. **Conclusions**: CBS (with either RYGB or SADI-S) results in a reduction in LDL-C, in contrast to the initial surgery with SG. However, CBS does not appear to provide additional benefits over SG in terms of other cardiometabolic parameters.

## 1. Introduction

Obesity is a global health concern due to its rising prevalence [1] and strong association with all cause-mortality, being the leading cause of death amongst the cardiovascular diseases [2]. Amongst the obesity-associated comorbidities, one of the most prevalent is dyslipidaemia [3], which is a major contributor to cardiovascular mortality, with elevated low-density lipoprotein cholesterol (LDL-C) playing a crucial role in atherogenesis.

Bariatric surgery is the most effective treatment for severe obesity and has proven to be better than other treatments in terms of weight loss, remission of associated comorbidities, and mortality reduction [4]. There are two predominant bariatric techniques: sleeve gastrectomy (SG) and Roux-en-Y gastric bypass (RYGB). Both techniques showed similar results in terms of weight loss and type 2 diabetes mellitus remission in the short term [5], but RYGB has shown greater efficacy in lowering LDL-C compared to purely restrictive procedures like SG [6]. This finding was confirmed in the BASALTO study, a randomized controlled trial conducted by our group, which demonstrated higher remission rates of elevated LDL-C one year after RYGB compared to SG (76% vs. 32%) [7]. Based on these findings, LDL-C should be considered when determining the most appropriate bariatric surgery procedure [8].

SG is currently the most widely performed procedure globally, surpassing RYGB, mainly due to its technical ease and favorable short-term outcomes [9]. Conversion bariatric surgery (CBS) may be required after SG in two main scenarios. Most commonly, it is indicated during follow-up due to suboptimal weight loss, the recurrence of comorbidities, or complications such as gastroesophageal reflux disease (GERD) or dysphagia caused by organic or functional stenosis. In these cases, the preferred conversion is typically to RYGB [10]. Alternatively, CBS can be planned in advance as part of a two-stage strategy in patients with very high BMI (usually > 50–60 kg/m^2^) or increased surgical risk. In this context, SG is performed as a first step to reduce the operative complexity and improve the metabolic status, followed by a hypoabsorptive procedure such as SADI-S once the patient is clinically stabilized. It is important to note that elevated LDL-C levels alone are not a recognized indication for conversion surgery. The current guidelines do not support dyslipidemia as a standalone criterion for revisional bariatric procedures.

Despite the growing use of CBS, its impact on the evolution of cardiometabolic parameters, including LDL-C, remains insufficiently studied. With the available evidence, we hypothesize that SG will not have a significant effect on LDL-C levels. However, after the CBS (either RYGB or SADI-S), LDL-C levels will decrease, as has been observed when these procedures are performed as initial bariatric surgeries. This reduction is expected to be more pronounced in the SADI-S group due to its greater hypoabsorptive effect. Our hypothesis is that these parameters—such as HDL cholesterol, triglycerides, HbA1c or blood pressure—will improve after SG, with further improvement or stabilization following the CBS.

The primary objective of this study is to assess the evolution of LDL-C concentrations levels in patients who undergo SG followed by a CBS (either RYGB or SADI-S), comparing the changes observed in the first procedure (SG) versus the second one (CBS). Secondary objectives include evaluating remission of high LDL-C and the evolution of additional cardiometabolic and anthropometric parameters before and after SG and CBS. Moreover, the study aims to compare the impact of the two CBS procedures (RYGB vs. SADI-S) on LDL-C levels.

## 2. Materials and Methods

### 2.1. Design and Study Population

A retrospective analysis was conducted of a non-randomized prospective cohort of patients with severe obesity undergoing bariatric surgery at the Hospital del Mar, Barcelona, from January 2016 to December 2023. Only patients who initially underwent SG and later required a CBS (RYGB or SADI-S) were included. The inclusion criteria for bariatric surgery included age between 18 and 65 years and meeting the National Institutes of Health (NIH) criteria for bariatric surgery at the time of the primary procedure: BMI > 40 kg/m^2^ or BMI > 35 kg/m^2^ with obesity-related comorbidities (type 2 diabetes, hypertension, dyslipidemia, or obstructive sleep apnea). The exclusion criteria for bariatric surgery included psychiatric or physical conditions contraindicating surgery, alcohol or substance dependency, pregnancy or lactation, poor general health preventing safe anesthesia and surgery, and a lack of commitment to long-term follow-up. Patients who did not complete at least one year of follow-up after the conversion surgery were also excluded from the study.

Patients from the OBEMAR prospective registry were included. Informed consent for both the procedure and participation in the registry was obtained from all patients. The study was approved by the Ethics Commission of the institution (CEIC IMIM Parc de Salut Mar reference number 2024/11854).

### 2.2. Outcomes Measures

The same endocrinologist evaluated all patients preoperatively and at months 3, 6, and 12 and then annually for both the first surgery with a SG and the CBS. However, for this study, only four time points were used for analysis: pre-SG (baseline), 12 months post-SG, pre-CBS, and 12 months post-CBS. The evaluation included biometric measurements (weight, blood pressure), laboratory tests [glucose, hemoglobin A1c, (HbA1c) total cholesterol, LDL-C, high-density lipoprotein cholesterol (HDL-C), insulin, triglycerides (TGs), kidney, and liver function].

The outcomes regarding metabolic and anthropometric (BMI and % total weight loss) values and remission of obesity comorbidities are defined by the Standardized Outcomes Reporting in Metabolic and Bariatric Surgery [11]. High LDL-C was defined as LDL-C ≥ 130 mg/dL or use of lipid-lowering therapy. Remission of high LDL-C was defined by postoperative LDL-C < 130 mg/dL without cholesterol-lowering drugs, as previously described [7]. Routine blood tests were performed after a 12-hour overnight fast at the hospital’s reference laboratory. Total cholesterol, LDL cholesterol, HDL cholesterol, and triglycerides were measured using enzymatic methods (Roche Cobas 8000 c701, Roche Diagnostics GmbH, Germany).

### 2.3. Surgical Techniques

The decision regarding the choice of surgical technique, both for the initial and conversion procedures, was made collaboratively in a multidisciplinary committee, which considered the patient’s clinical characteristics and comorbidities, as well as the recommendations established in international guidelines [12] and local recommendations (PADEICS, Programes Assistencials d’Expertesa de l’Institut Català de la Salut) [13].

According to institutional criteria based on the PADEICS guidelines, SG was generally preferred as the primary bariatric procedure in patients with a BMI < 40 kg/m^2^ without metabolic syndrome or severe gastroesophageal reflux (esophagitis grade II–III). In patients with a BMI between 40 and 50 kg/m^2^, RYGB was the procedure of choice when metabolic syndrome and/or significant GERD were present, while SG could still be considered in young, active patients without these conditions. For patients with BMI > 50 kg/m^2^, malabsorptive procedures such as SADI were indicated. These could be performed either as a single-stage surgery (SADI-S) or as a planned two-stage approach—starting with SG and completing the malabsorptive component (SADI) 12–18 months later—especially in cases with BMI > 60 kg/m^2^ or when the surgical risk was high [12,13].

There are three main scenarios that may require CBS after SG. The first and most frequent is the development of complications such as GERD or dysphagia secondary to gastric stenosis. The second includes patients with an inadequate weight loss response—defined as <50% excess weight loss (EWL) or a persistent body mass index (BMI) >35 kg/m^2^—or with poor control of obesity-related comorbidities. In these cases, conversion to RYGB or SADI-S may be considered depending on the patient’s initial BMI and comorbidity profile. The third scenario corresponds to patients undergoing a planned two-stage procedure, such as SADI-S, as previously described according to PADEICS. It should be emphasized that elevated LDL-C is not among the recognized indications for conversion surgery. In our cohort, no patients underwent CBS for dyslipidemia alone, and all conversions were based on established clinical criteria.

SG involved the removal of 60–80% of the stomach, preserving a vertical gastric sleeve along the lesser curvature with an approximate capacity of 150 mL [14]. Conversion from SG to RYGB consisted of a 150 cm antecolic Roux limb with a linear stapled gastrojejunostomy and the creation of a 50 mL gastric pouch, along with the exclusion of 60–100 cm of proximal jejunum. SADI-S was performed on a stomach previously treated with SG and involved a duodeno–ileal anastomosis located 300 cm from the ileocecal valve [10]. All procedures were performed laparoscopically by the same bariatric surgery team.

### 2.4. Sample Size

Assuming an alpha risk of 0.05 and a beta risk below 0.2 in a bilateral contrast, a minimum of 21 subjects were required to detect a difference of at least 20 mg/dL in LDL-C reduction comparing the pre- and one-year-post-SG periods versus the pre- and one-year-post-CBS periods. Based on data from the BASALTO study, we assumed a standard deviation of 32 mg/dL in LDL-C changes [7].

### 2.5. Statistical Analysis

Continuous variables are expressed as mean ± standard deviation if normally distributed, or as median with interquartile range if non-normally distributed. Categorical variables are reported as percentages and frequencies. Normality was assessed using the Kolmogorov–Smirnov test, and skewed variables were log-transformed if necessary. Student’s *t*-test was used to compare normally distributed continuous variables, while chi-square and Fisher’s exact tests were used for categorical variables. To evaluate LDL-C changes, four time points were considered: before and one year after SG, and before and one year after the second surgery. The change in LDL-C concentration in the SG period was compared to the change in the CBS period. Patients under cholesterol-lowering treatment at baseline or at the 12-month follow-up were excluded from the analysis of LDL-C level changes after bariatric surgery. Similar analyses were applied to the other cardiometabolic variables. For the evolution of continuous variables over time, repeated-measures ANOVA was used. To compare the magnitude of change between the SG and CBS periods, a paired *t*-test was applied. A *p*-value < 0.05 was considered statistically significant. Data were analyzed using SPSS Statistics software (version 25 for Windows; SPSS, Chicago, IL, USA).

## 3. Results

### 3.1. Description of the Cohort and Baseline Characteristics

A total of 28 patients underwent CBS during the study period and met the inclusion criteria, including 16 (57%) who received RYGB and 12 (43%) who underwent SADI-S. No losses to follow-up were recorded during the first year after CBS, nor among patients scheduled for a two-stage SADI approach. Amongst the 16 RYGB-treated patients, 12 (75%) were indicated for GERD, 1 (6.25%) for poor weight and metabolic control, and 3 (18.75%) for other SG complications. Of the 12 SADI-S-treated patients, 10 (83.3%) were indicated as a planned two-stage SADI-S, 2 (16.6%) for poor weight and metabolic control, and 1 for other reasons (8.3%). The mean time between SG and CBS was 93.5 ± 45.3 months for RYGB and 31.0 ± 45.2 months for SADI-S (*p* < 0.001). The prior SG characteristics of the study population are shown in Table 1. No differences between groups were observed except for BMI, which was higher in the SADI group.

The baseline characteristics of the entire cohort, as well as those of the two main subgroups—patients undergoing RYGB and patients undergoing SADI—were recorded. Continuous variables are expressed as mean ± standard deviation if normally distributed, or as median with interquartile range if non-normally distributed. Categorical variables are reported as percentages and frequencies. The *p*-value indicates differences between groups (<0.05 was considered statistically significant).

### 3.2. Weight Loss

**Figure 1:**
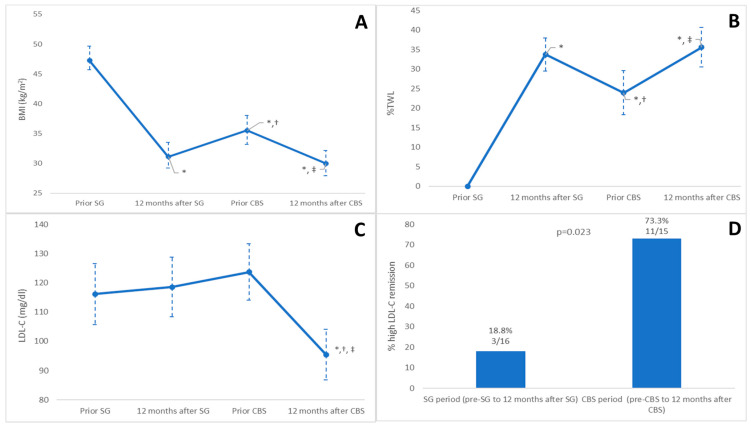
(**A**): Evolution of BMI in the entire cohort throughout the follow-up. (**B**): Evolution of TWL% in the entire cohort throughout the follow-up. (**C**): Changes in LDL cholesterol in the entire cohort during the follow-up. (**D**): Remission of high LDL-C in both time periods. Data are expressed as the means with 95% confidence intervals for (**A**–**C**). Data are expressed as % for (**D**). BMI: Body Mass Index; SG: Sleeve Gastrectomy; CBS: Conversion Bariatric Surgery; RYGB: Roux-en-Y Gastric Bypass; SADI: Single-Anastomosis Duodeno–Ileal Bypass. * Significant change compared to baseline (prior SG) (*p* < 0.05). † Significant change compared to 12 months after SG (*p* < 0.05). ‡ Significant change compared to prior CBS (*p* < 0.05).

Patients experienced a significant BMI reduction during the first 12 months after SG, a significant increase in the interval between surgeries, and a further significant weight loss in the year following CBS. Weight loss was significantly greater in the post-SG period compared to the post-CBS period (Figure 1A). The %TWL showed a similar evolution (Figure 1B).

### 3.3. Primary Outcome: LDL-C Concentration Changes

Changes in LDL-C concentration during follow-up were assessed in the 23 patients who were not taking cholesterol-lowering drugs at baseline. As shown in Figure 1C and Table 2, the LDL-C levels remained stable 12 months after SG but showed a significant reduction in the year following the CBS procedure.

### 3.4. Secondary Outcomes

#### 3.4.1. Remission of High LDL-C

Before SG, 16 patients (57.1% of the total) met the criteria for high LDL-C. Of these, three (18.8%) achieved remission twelve months after SG. Before the CBS, 15 patients (53.6% of the total) still had high LDL-C. After the CBS, 11 out of 15 patients (73.3%) achieved remission of high LDL-C (*p* = 0.023) (Figure 1D).

#### 3.4.2. Remission of Obesity-Associated Comorbidities

Twelve months after SG, remission of type 2 diabetes mellitus was 100% (12 of 12), remission of hypertension was 63.6% (14 of 22), and remission of hypertriglyceridemia was 100% (6 of 6). Before CBS there were no patients with type 2 diabetes mellitus, while nine patients had hypertension, of which none remitted (0%), and three patients had hypertriglyceridemia (two relapsed in between surgeries and one appeared de novo), of which 100% (three of three) remitted in the 12 months of follow-up.

The evolution of biochemical parameters associated with obesity is shown in Table 2. Total cholesterol followed a similar trend to LDL-C over time. Significant differences between the SG and CBS periods were observed in several parameters, including HDL-C, triglycerides, HbA1c, diastolic blood pressure, and GGT. These differences were mainly driven by a marked improvement in the cardiometabolic profile during the SG period, followed by maintenance or only non-significant additional changes during the CBS period. In contrast, liver enzymes GPT and GOT showed a different behavior, with a significant increase being observed after CBS.

The values at each follow-up time point (baseline, 12 months after SG, before CBS, and 12 months after CBS) are expressed as means ± standard deviations. Changes over the different follow-up periods (pre-SG and 12 months after SG; pre-CBS and 12 months after CBS) were expressed as means with 95% confidence intervals. ANOVA models with Bonferroni adjustment were used to analyze the evolution of continuous variables in each group and assess differences between the groups from baseline. The last column expresses the statistical significance of the changes between baseline and 12 months after SG compared to changes between pre-CBS and 12 months after CBS, using paired *t*-tests.

#### 3.4.3. Differences Between Techniques (RYGB vs. SADI-S)

**Figure 2:**
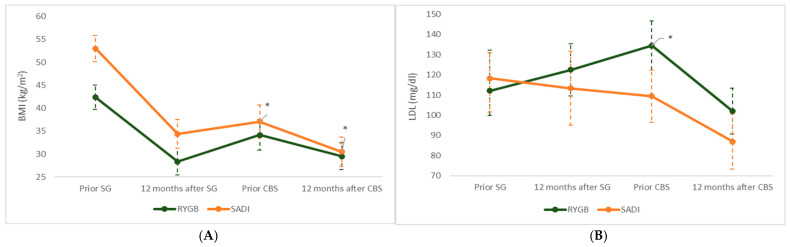
(**A**): Evolution of BMI in the two main groups throughout the follow-up. (**B**): Changes in LDL cholesterol during the follow-up in the group undergoing a RYGB and the group undergoing SADI. Data are expressed as the means with 95% confidence intervals. BMI: Body mass index; SG: sleeve gastrectomy; CBS: conversion bariatric surgery; RYGB: Roux-en-Y gastric bypass; SADI-S: single-anastomosis duodeno–ileal bypass with sleeve gastrectomy. * Significant difference between techniques in the change from baseline (pre-SG) at the corresponding time point (*p* < 0.05).

Differences between the two conversion techniques were observed in the evolution of both BMI (Figure 2A) and LDL cholesterol (Figure 2B). Although both RYGB and SADI-S led to a similar magnitude of LDL-C reduction during the CBS period, their overall trajectories differed. Specifically, patients in the RYGB group showed a marked increase in LDL-C concentrations in the period prior to conversion, a pattern that was not observed in the SADI-S group. Regarding BMI, significant differences were found both before and 12 months after CBS, with consistently higher decreases in patients undergoing SADI-S.

The remaining parameters showed a similar evolution during follow-up in both the RYGB and SADI-S groups, consistent with the pattern that was observed in the overall cohort: a marked improvement during the SG period, followed by maintenance of the achieved benefits during the CBS period. In contrast, GOT and GPT exhibited a distinct trajectory, with significantly greater increases being observed during the CBS period in patients undergoing SADI-S compared to those converted to RYGB (Supplementary Table S1).

## 4. Discussion

This study provides novel data on the evolution of LDL-C in patients undergoing CBS. Our findings confirm the initial hypothesis: SG, when performed as a primary procedure, has little impact on LDL-C levels, whereas both RYGB and SADI-S lead to a significant reduction after conversion. There is solid evidence supporting the superiority of both RYGB and SADI-S over SG in lowering LDL-C when used as primary procedures [6,10], and our results extend this benefit to the conversion setting, demonstrating that switching from SG to either of these hypoabsorptive techniques also has a positive effect on LDL-C.

The superiority of techniques with a hypoabsorptive component in reducing LDL-C concentrations does not yet have a well-established explanation. The effect of LDL-C reduction has long been described as independent of weight loss [15] and associated instead with the surgical technique performed. Moreover, the BASALTO randomized clinical trial showed that LDL-C levels improve more significantly after RYGB compared to SG, even when both procedures achieve similar levels of weight loss [7]. This led to the hypothesis that decreased intestinal absorption is the main explanation for the differences observed between purely restrictive and hypoabsorptive procedures [16,17]. Supporting this hypothesis is the study by Pihlajamäki et al. [18], which assessed cholesterol absorption markers one year after surgery in patients who underwent RYGB and compared them to those who had gastric banding (a now obsolete restrictive technique). This study found that while cholesterol synthesis markers decreased in both groups, a reduction in cholesterol absorption markers was only observed after RYGB and not after gastric banding [18]. Although the baseline LDL-C values in our cohort were close to the upper limit of the normal range, the observed reduction following CBS remains clinically relevant given the elevated cardiovascular risk profile of patients with severe obesity. It should be noted that LDL-C levels alone were not the basis for surgical decision-making. The added benefit on LDL-C observed in our study is clinically relevant but occurred in patients whose indications for conversion were unrelated to lipid control. Furthermore, in patients with elevated LDL-C after SG, lipid-lowering pharmacological treatment (e.g., statins) should be considered before or alongside surgical options, especially when CBS is not otherwise indicated.

Beyond LDL-C, weight and other metabolic outcomes showed a different pattern. A slight increase in BMI and a modest decrease in %TWL were observed between 12 months post-SG and the time of conversion. However, this modest weight regain was minimal, and most patients maintained clinically meaningful weight loss at the time of CBS. Notably, CBS helped partially restore previous weight loss in some cases, which may represent an additional clinical benefit for selected patients seeking improved weight control. These results must be interpreted in light of our cohort’s profile, where the main indications for conversion were GERD and planned two-stage SADI-S. In contrast, studies where CBS is primarily indicated due to weight regain tend to report a more marked reversal in weight parameters before conversion.

Similarly, SG alone achieved high remission rates of major obesity-related comorbidities in our cohort—including type 2 diabetes, hypertriglyceridemia, and hypertension—with accompanying improvements in HDL-C, HbA1c, diastolic blood pressure, and GGT. These cardiometabolic improvements remained stable after CBS, without further gains. This contrasts with studies in which CBS was performed for inadequate weight or metabolic control, where conversion led to significant remission of comorbidities. For example, Osorio et al. reported remission rates after SADI-S of 44.4% for diabetes and 36.4% for hypertension [19], while Sánchez-Pernaute et al. observed even higher rates—94% for diabetes and 56% for hypertension [20]. Other cohorts, such as those of Wilczynski M, or Al-Sabah S, also exhibited variable but often substantial metabolic improvements after CBS [21,22].

Our findings are particularly relevant given the growing prevalence of the patient profile represented in our cohort. With SG now being established as the most widely performed bariatric procedure worldwide and GERD as a frequent long-term complication, as well as the increasing adoption of two-stage SADI-S in patients with severe obesity [9,10], conversions that are not driven by metabolic failure are becoming increasingly common. Although the study was not designed to compare outcomes between different indications for conversion, the predominance of these two profiles (GERD and planned two-stage SADI-S) provides a representative view of this emerging clinical scenario. In this evolving clinical scenario, our results suggest that CBS continues to provide clear added value: it contributes to an additional reduction in LDL-C—a key cardiovascular risk factor—and offers a modest but meaningful increase in weight loss beyond that achieved with SG alone, while preserving the metabolic improvements already attained.

We initially hypothesized that SADI-S would lead to a greater reduction in LDL-C due to its higher hypoabsorptive potential compared to RYGB. However, this could not be confirmed. Although the LDL-C trajectories differed between groups, this was mainly driven by a preoperative increase in LDL-C in the RYGB group—likely related to the longer interval since SG (93.5 vs. 31.0 months). Despite these differences, both CBS techniques achieved a comparable reduction in LDL-C after surgery. Nonetheless, the limited sample size and baseline differences between groups prevent definitive conclusions.

In relation to the other parameters, no differences were observed between the groups. This finding is consistent with previous evidence. Thomopoulos et al. reported in a meta-analysis [23] comparing RYGB vs. SADI-S as CBS in the medium–long term that differences are only found in weight loss (superior in SADI) and in the resolution of GERD and other functional issues after SG (more significant in RYGB). The results in terms of the resolution of comorbidities during the long-term follow-up were similar [23]. These results contradict those presented by Salama et al. in another meta-analysis [24], with a higher remission of comorbidities (especially diabetes mellitus and hypertension) in the SADI-S group [24]. In our cohort, patients undergoing SADI started from a significantly higher BMI than those undergoing RYGB yet achieved similar BMI values at 12 months post-CBS. This convergence may reflect a stronger weight loss effect of SADI in patients with more severe obesity. While overall metabolic outcomes were comparable between groups, some parameters—such as HbA1c and triglycerides—only improved in the SADI group, supporting its potential benefit in selected high-risk profiles. These findings contribute to the growing evidence supporting the utility of SADI in patients with very high BMIs.

A moderate increase in GOT and GPT at 12 months after CBS was observed, being more pronounced in patients undergoing SADI-S than in those with RYGB. In hypoasbortive procedures, alterations in the hepatic profile have been described, consisting of a transient elevation of enzymes for months after surgery, but with normalization at one year of follow-up [25,26]. Further investigation is needed to assess these changes.

The main limitations of this study are that it is a unicentric retrospective study, and that the CBS can be indicated for different medical reasons, which can affect the results. The follow-up was limited to one year post-CBS, and only patients with complete follow-up after SG were included. In addition, the sample size was small, and there was a marked difference in the time interval between SG and CBS across the two surgical techniques, which may have affected the metabolic status at the time of conversion. Moreover, the study likely lacks sufficient power to detect differences in analytical parameters beyond LDL-C or to adequately compare the two CBS subgroups. Finally, the study is also limited by possible baseline differences between CBS subgroups (RYGB vs. SADI), especially regarding cardiometabolic parameters, which may have influenced the observed outcomes, despite the lack of statistical significance.

## 5. Conclusions

In a cohort where CBS was primarily indicated for gastroesophageal reflux disease or as part of a planned two-stage SADI—rather than for weight regain or metabolic failure—CBS did not lead to further improvements in most cardiometabolic parameters beyond those already achieved with SG. However, it did result in a consistent and clinically relevant reduction in LDL-C levels, one of the main biochemical markers of cardiovascular risk. The metabolic effects observed support the increasing use of SADI as a conversion procedure.

## Figures and Tables

**Table 1:** Pr SG characteristics of the patients included in the study.

	Total Cohort (*n* = 28)	RYGB (*n* = 16)	SADI-S (*n* = 12)	*p*-Value
Sex, *n* (% females)	19 (67.9%)	13 (81.3)	6 (50)	0.114
Age (years), mean ± SD	44.5 ± 9.3	46.6 ± 8.7	41.6 ± 9.7	0.08
Weight (kg), mean ± SD	133.3 ± 24.1	113.7 ± 15.4	153.7 ± 19.1	<0.001
BMI (kg/m^2^), mean ± SD	46.8 ± 7.3	42.3 ± 4.3	53.4 ± 19.1	<0.001
Smoking habit, *n* (%)	10 (35.7%)	3 (18.8)	7 (58.3)	0.05
Patients with HTA, *n* (%)	22 (78.6%)	13 (81.3)	9 (75)	1
HTA pharmacological treatment, *n* (%)	17 (60.7%)	9 (56.3)	8 (66.7)	0.705
Systolic BP (mmHg), mean ± SD	133 ± 21.6	134.3 ± 25.5	131.3 ± 15.3	0.366
Dyastolic BP (mmHg), mean ± SD	85.6 ± 11	87.4 ± 11.3	83 ± 10.7	0.161
Patients with high LDL-C, *n* (%)	16 (57.1%)	9 (56.3)	7 (58.3)	1
Patients with statins, *n* (%)	5 (17.9%)	4 (25)	1 (8.3)	0.355
Total cholesterol (mg/dL), mean ± SD	187.7 ± 28.2	190.6 ± 31.5	183.8 ± 23.8	0.271
LDL-C (mg/dL), median (IQ range)	125.4 (102–133)	125.4 (85.1–134.8)	123 (98.1–131.3)	0.816
HDL-C (mg/dL), median (IQ range)	46.3 (41.8–53.3)	46.5 (41.3–52)	45.75 (38.3–62.3)	0.996
Hypertriglyceridemia, *n* (%)	6 (21.4%)	5 (31.3)	1 (8.3)	0.196
Patients with fibrate therapy, *n* (%)	1 (3.6%)	0 (0)	1 (8.3)	0.429
Triglycerides (mg/dL), mean ± SD	123.1 ± 52.6	124.5 ± 51.7	113.3 ± 47.9	0.282
Patients with type 2 diabetes, *n* (%)	6 (21.4%)	3 (18.8)	3 (25)	1
Glycaemia (mg/dL), median (IQ range)	96.5 (91.5–105.3)	95 (87.8–105.8)	103.5 (93–110.1)	0.236
HbA1c (%), mean ± SD	5.6 ± 0.8	5.6 ± 0.9	5.6 ± 0.6	0.464
Insulin (mcIU/mL), median (IQ range)	15 (9–22.8)	11.6 (9.8–17.6)	17.3 (9.5–44)	0.120
GOT (U/L), mean ± SD	20.6 ± 7.9	20.8 ± 8.5	20.2 ± 7.4	0.422
GPT (U/L), mean ± SD	24.6 ± 13.7	24.2 ± 15	25.2 ± 12.2	0.429
GGT (U/L), median (IQ range)	24.5 (15.8–47.8)	22 (13.5–38.8)	26.50 (20.5–50.3)	0.291

RYGB: Roux-en-Y gastric bypass; SADI-S: single-anastomosis duodeno–ileal bypass with sleeve gastrectomy; BMI: body mass index; HTA: arterial hypertension; BP: blood pressure; LDL-C: low-density lipoprotein cholesterol; HDL-C: high-density lipoprotein cholesterol; HbA1c: glycated hemoglobin.

**Table 2:** Evolution of biochemical and anthropometric parameters at baseline and 12 months after both surgeries.

Parameter	SG Period			CBS Period			Between-Period Difference
	Baseline (1)	12 Months (2)	Δ (1→2) (Mean, 95% IC)	Prior to CBS (3)	12 Months Post-CBS (4)	Δ (3→4) (Mean, 95% IC)	Δ (1→2) vs. Δ (3→4)
LDL-C (mg/dL)	115.2 ± 25.4	118.9 ± 27.9	3.3 (−13.6 to 20.1)	123.7 ± 25	97.7 ± 22.1 *†‡	−25.7 (−37.5 to −13.9)	<0.001
Cholesterol (mg/dL)	185.8 ± 24.2	194.3 ± 31.4	8.2 (−8.2 to 24.5)	198.2 ± 27	169 ± 22.9 *†‡	−29.3 (−43.2 to 15.4)	<0.001
HDL cholesterol (mg/dL)	50.2 ± 13.6	67 ± 32.8 *	15.9 (1 to 30.8)	58.8 ± 12.4 *	60.1 ± 14.2 *	1.1 (−4.7 to 7)	<0.001
Triglycerides (mg/dL)	119.7 ± 49.5	78.8 ± 36.9 *	−41.1 (−73.1 to −9.2)	96.9 ± 42.4	84.9 ± 42.2 *	−13.6 (−39.7 to 12.5)	0.005
Glucose (mg/dL)	109.2 ± 51.4	89 ± 10.5	−18.9 (−46.1 to 8.3)	88.5 ± 9.6	89.6 ± 10.5	1 (−3.7 to 5.9)	0.199
HbA1c (%)	5.6 ± 0.8	5.2 ± 0.4	−0.4 (−0.8 to 0.03)	5.2 ± 0.3 *	5.2 ± 0.3 *	−0.1 (−0.2 to 0.1)	0.022
HOMA	5.7 ± 6.9	2.1 ± 2	−3.7 (−8.2 to 0.8)	2.1 ± 1.3	1.7 ± 1.1	−0.4 (−1.4 to 0.6)	0.069
Systolic BP (mmHg)	132.8 ± 22.6	122.5 ± 20.5	−9 (−22.3 to 4.3)	124.6 ± 19.4	129.5 ± 17.3	3.9 (−5.2 to 13)	0.116
Diastolic BP (mmHg)	85.3 ± 10.8	75 ± 9.4 *	−9.2 (−17.3 to −1.2)	76.2 ± 9.3	78.9 ± 9.7	2.5 (−4.1 to 9.1)	0.032
GOT (U/L)	20.8 ± 8	18.4 ± 4.9	−2.3 (−7.4 to 2.7)	17 ± 4	27.8 ± 10.8 *†‡	11.4 (6.4 to 16.4)	<0.001
GPT (U/L)	24.6 ± 13. 7	17.6 ± 7.9 *	−7.2 (−13.5 to −0.8)	14.6 ± 5 *	33.5 ± 23.2 †‡	20.6 (9.6 to 31.6)	<0.001
GGT (U/L)	30.6 ± 18.9	21.5 ± 13.7	−9.2 (−18.5 to 0.1)	20.9 ± 12.5 *	23.5 ± 17.3	2.8 (−3.7 to 9.3)	0.012
BMI (kg/m^2^)	47.3 ± 7.2	31.1 ± 6 *	−16.3 (−19.6 to −13)	35.5 ± 6.1 *†	30 ± 5.2 *‡	−5.6 (−7.7 to −3.5)	<0.001

SG: sleeve gastrectomy; CBS: conversion bariatric surgery. * Significant change compared to baseline (prior SG, 1) (*p* < 0.05). † Significant change compared to 12 months after SG (2) (*p* < 0.05). ‡ Significant change compared to prior CBS (3) (*p* < 0.05).

## Data Availability

The data that support the findings of this study are not publicly available due to privacy and ethical restrictions but may be made available from the corresponding author upon reasonable request and approval by the institutional ethics committee.

## Supplementary Materials

The following supporting information can be downloaded at: https://www.mdpi.com/article/10.3390/jcm14144901/s1, Table S1: Evolution of biochemical and anthropometric parameters at baseline and 12 months after both surgeries for both CBS groups (RYGB and SADI-S).

## Abbreviations

The following abbreviations are used in this manuscript:

CBS	Conversion bariatric surgery
SG	Sleeve gastrectomy
RYGB	Roux-en-Y gastric bypass
SADI-S	Single-anastomosis duodeno–ileal bypass with sleeve gastrectomy
LDL-C	Low-density lipoprotein cholesterol
GERD	Gastroesophageal reflux disease
