# Supplementary material for: Bariatric Conversion Surgery Impact on LDL Cholesterol in Patients Previously Treated with Sleeve Gastrectomy
Source: J Clin Med. 2025 Jul 10;14(14):4901. doi: 10.3390/jcm14144901 (PMC12295971; doi:10.3390/jcm14144901)
Supplement: Supplementary file 1 [file jcm-14-04901-s001.zip › jcm-3726217 Supplementary Table S1.pdf]

Supplementary Table S1. Evolution of biochemical and anthropometric parameters at baseline and 12 months after both surgeries for both CBS groups (RYGB and SADI-S).

Parameter	CBS technic	SG period			CBS period			Between-period difference	Difference Between SADI and RYGB during follow-up
		Baseline (1)	12 months (2)	∆ (1 →2)	Prior CBS (3)	12 months post-CBS (4)	∆ (3 →4)	∆ (1→2) vs ∆ (3→ 4)	
LDL cholesterol (mg/dL)	RYGB	110.1 ± 30.8	122.2 ± 27.1	12.1 (-11.2 to 35.5)	134.9 ± 24.3*	104.9 ± 22.7‡	-30 (-46.3 to -13.7)	<0.001	0.009
	SADI-S	120.8 ± 17.6	115.2 ± 29.6	-5.6 (-30 to 18.7)	111.4 ± 20.1	89.9 ± 19.5*†‡	-21 (-38.4 to -4.5)	<0.001	
Cholesterol (mg/dl)	RYGB	183.1 ± 29.6	199.4 ± 27.9	16.3 (-6.3 to 39)	207.8 ± 24.7*	180 ± 17‡	-27.8 (-47 to -8.6)	<0.001	0.036
	SADI-S	188.7 ± 17.5	188.7 ± 35.3	0 (-23.6 to 23.6)	187.8 ± 26.5	157 ± 23*†‡	-30.8 (-50.9 to -10.8)	<0.001	
HDL cholesterol (mg/dl)	RYGB	49.6 ± 11.7	72 ± 40.2*	22.4 (2.9 to 41.8)	59.4 ± 12.1*	62.4 ± 14.3*	3 (-4.7 to 10.7)	<0.001	0.511
	SADI-S	50.9 ± 16.3	60.3 ± 19	9.5 (-13 to 31.9)	57.9 ± 13.2	57.2 ± 14	-0.8 (-9.6 to 8.1)	0.057	
Triglycerides (mg/dl)	RYGB	124.5 ± 51.7	84.9 ± 42.4	-39.6 (-81.5 to 2.3)	103.3 ± 40.4	100.7 ± 47.9	-2.6 (-36.8 to 31.5)	0.097	0.538
	SADI-S	111.3 ± 48	70.7 ± 27.7	-42.7 (-91 to 5.7)	88.4 ± 45.2	63.9 ± 20.1*	-24.5 (-64 to 15)	0.025	
Glucose (mg/dL)	RYGB	114.3 ± 68	86.6 ± 11.5	-27.8 (-63.4 to 7.9)	87.8 ± 9.4	89.6 ± 12.6	1.8 (-4.5 to 8.1)	0.081	0.607

	SADI-S	102.3 ± 9.4	92.3 ± 8.5	-10.1 (-51.1 to 31.1)	89.4 ± 10.3	89.7 ± 7.3	0.3 (-7 to 7.5)	0.603	
HbA1c (%)	RYGB	5.6 ± 0.9	5.4 ± 0.4	-0.2 (-0.7 to 0.3)	5.3 ± 0.3	5.3 ± 0.3	0 (-0.2 to 0.2)	0.147	0.266
	SADI-S	5.6 ± 0.6	5.1 ± 0.3	-0.6 (-1.1 to 0.1)	5.2 ± 0.4	5 ± 0.3*	-0.1 (-0.4 to 0.1)	0.045	
HOMA	RYGB	5.5 ± 8.5	2.3 ± 2.6	-3.2 (-9 to 2.5)	2.3 ± 1.6	1.8 ± 1.3	-0.5 (-1.7 to 0.8)	0.197	0.970
	SADI-S	6 ± 4.2	1.8 ± 0.9	-4.2 (-11.1 to 2.7)	1.9 ± 1	1.6 ± 0.6	-0.3 (-1.8 to 1.2)	0.323	
Systolic BP (mmHg)	RYGB	133.9 ± 26.4	119.9 ± 24.2	-14 (-30.3 to 2.3)	124.9 ± 22.3	132.5 ± 18.5†	7.6 (-3.6 to 18.8)	0.008	0.369
	SADI-S	130.8 ± 15.7	126.8 ± 2	-4 (-25.1 to 17.1)	124.1 ± 14.5	124.3 ± 14.6	0.2 (-14.2 to 14.6)	0.878	
Diastolic BP (mmHg)	RYGB	87.5 ± 11.6	74.1 ± 11.3*	-13.5 (-23.3 to -3.6)	76.3 ± 10.7*	79.6 ± 10.5	3.3 (-4.7 to 11.4)	0.006	0.346
	SADI-S	81.4 ± 8.4	76.4 ± 5.3	-5 (-17.7 to 7.7)	76.1 ± 7	77.8 ± 8.7	1.7 (-8.8 to 12.1)	0.742	
GOT (U/L)	RYGB	21.2 ± 8.6	18.4 ± 5.9	-2.8 (-9.3 to 3.8)	16.6 ± 4.5	23.7 ± 5.9‡	7.1 (0.6 to 13.6)	0.003	0.019
	SADI-S	20.2 ± 7.4	18.3 ± 3.3	-1.9 (-9.6 to 5.8)	17.6 ± 3.5	33.4 ± 13.4*†‡	15.7 (8.1 to 23.3)	<0.001	
GPT (U/L)	RYGB	24.2 ± 15	18.1 ± 9.3	-6.1 (-14.2 to 2)	13.2 ± 3.1*†	24.6 ± 11.3	11.4 (-2.7 to 25.5)	0.003	0.003

	SADI-S	25.2 ± 12.2	17 ± 5.8	-8.2 (-18 to 1.6)	16.7 ± 6.6	46.5 ± 29.8*†‡	29.8 (12.8 to 46.8)	<0.001	
GGT (U/L)	RYGB	27 ± 16.9	19.2 ± 11.5	-7.8 (-20.4 to 4.7)	18.8 ± 9.4	19.4 ± 10.4	0.7 (-8.1 to 9.4)	0.245	0.850
	SADI-S	34.9 ± 21.2	24.3 ± 16	-10.6 (-24.4 to 3.2)	23.4 ± 15.6	28.3 ± 22.7	4.9 (-4.7 to 14.5)	0.032	

SG: sleeve gastrectomy, CBS: conversion bariatric surgery, RYGB: Roux-en-Y Gastric Bypass, SADI-S: single anastomosis duodeno-ileal bypass with sleeve gastrectomy

The values at each follow-up time point (baseline, 12 months after SG, before CBS, and 12 months after CBS) are expressed as means ± standard deviations. Changes over the different follow-up periods (pre-SG and 12 months after SG; pre-CBS and 12 months after CBS) were expressed as means with 95% confidence intervals. ANOVA models with Bonferroni adjustment were used to analyze the evolution of continuous variables in each group and assess differences between the groups from baseline. The penultimate column expresses the statistical significance of the changes between baseline and 12 months after SG compared to changes between pre-CBS and 12 months after CBS using paired t-tests. The last column indicates whether there are statistically significant differences between the CBS procedures.

*Significant change compared to baseline (prior SG, 1) (p<0.05).

†Significant change compared to 12 months after SG (2) (p<0.05).

‡Significant change compared to prior CBS (3) (p<0.05).
